# 16S *rRNA* Terminal Restriction Fragment Length Polymorphism for the Characterization of the Nasopharyngeal Microbiota

**DOI:** 10.1371/journal.pone.0052241

**Published:** 2012-12-20

**Authors:** Silvio D. Brugger, Laurence Frei, Pascal M. Frey, Suzanne Aebi, Kathrin Mühlemann, Markus Hilty

**Affiliations:** 1 Institute for Infectious Diseases, University of Bern, Bern, Switzerland; 2 Department of General Internal Medicine, University Hospital of Bern, Bern, Switzerland; 3 Department of Infectious Diseases, University Hospital of Bern, Bern, Switzerland; Baylor College of Medicine, United States of America

## Abstract

A novel non-culture based 16S *rRNA* Terminal Restriction Fragment Length Polymorphism (T-RFLP) method using the restriction enzymes *Tsp509I* and *Hpy166II* was developed for the characterization of the nasopharyngeal microbiota and validated using recently published 454 pyrosequencing data. 16S *rRNA* gene T-RFLP for 153 clinical nasopharyngeal samples from infants with acute otitis media (AOM) revealed 5 *Tsp509I* and 6 *Hpy166II* terminal fragments (TFs) with a prevalence of >10%. Cloning and sequencing identified all TFs with a prevalence >6% allowing a sufficient description of bacterial community changes for the most important bacterial taxa. The conjugated 7-valent pneumococcal polysaccharide vaccine (PCV-7) and prior antibiotic exposure had significant effects on the bacterial composition in an additive main effects and multiplicative interaction model (AMMI) in concordance with the 16S *rRNA* 454 pyrosequencing data. In addition, the presented T-RFLP method is able to discriminate *S. pneumoniae* from other members of the Mitis group of streptococci, which therefore allows the identification of one of the most important human respiratory tract pathogens. This is usually not achieved by current high throughput sequencing protocols. In conclusion, the presented 16S *rRNA* gene T-RFLP method is a highly robust, easy to handle and a cheap alternative to the computationally demanding next-generation sequencing analysis. In case a lot of nasopharyngeal samples have to be characterized, it is suggested to first perform 16S *rRNA* T-RFLP and only use next generation sequencing if the T-RFLP nasopharyngeal patterns differ or show unknown TFs.

## Introduction

Previous studies on the nasopharyngeal microbiota have mainly relied on conventional culture identification of the major pathogens *Streptococcus pneumoniae*, *Haemophilus influenzae, Stahphylococcus aureus* and *Moraxella catarrhalis.* These bacteria colonize the human nasopharynx as a first step in the pathogenesis of acute otitis media (AOM) [1], [2], [3], [4]. However, selective analysis focusing only on a few pathogens may lead to an underestimation of the influence of a generally disturbed or altered nasopharyngeal microbiota on disease progression.

In recent years there has been significant development of non-cultural methods to characterize microbial communities of different human body sites [5], [6], [7], [8]. Among those, next-generation sequencing (e.g., 454 and Illumina pyrosequencing) seems to be the most appropriate technique for the future. It is based on the amplification of 16S *rRNA* gene which has been widely used as a bacterial classification marker [9]. Using primers specific for conserved border regions and subsequent analysis of the variable sections amplified in-between allows species identification. However, there is no standardized protocol for analyzing high throughput data and careful evaluation is needed. Among the parameters, which could have an influence on the outcome of the analysis are i) the choice of primers, ii) the DNA extraction protocols, iii) the amplification conditions, iv) the alignment of sequences and v) the Operational Taxonomic Unit (OTU) definition.

In a recent study, we investigated the influence of antibiotic consumption and vaccination with the conjugated 7-valent pneumococcal polysaccharide vaccine (PCV-7) on the nasopharyngeal microbiota in children with and without AOM using 454 16S *rRNA* sequencing [10]. Within this study, an alternative 16S *rRNA* Terminal Restriction Fragment Length Polymorphism (T-RFLP) method to characterize the microbiota of the nasopharynx was developed and validated. This novel approach offers the advantages of being quick, easy to handle and cheap.

## Materials and Methods

### 

#### Ethics statement

Samples used for this study were collected within a nationwide surveillance program from outpatients with acute otitis media, which is ongoing since 1998. This surveillance program is part of the governmental public health surveillance and is therefore exempted from approval by Institutional Review Boards.

### *In silico* Design of Terminal Restriction Fragment Length Polymorphism (T-RFLP)

We downloaded 38 16S *rRNA* sequences of bacteria reported to be characteristic for the nasopharynx [10] which were then trimmed in 4 different groups ranging from 8F-907, 8F-1391, 341–907 and 341–1391 (*E. coli* numbering). These groups were uploaded to the restriction Enzyme Picker website (http://rocaplab.ocean.washington.edu/tools/repk) to select enzymes which showed the highest possible discriminatory power. This website stores 16S *rRNA* sequences and shows a ranking and combination of enzymes for optimal bacterial 16S diversity coverage. Moreover, an emphasis was laid on the fact that the enzymes could distinguish the ‘commensal’ Mitis group members from *Streptococcus pneumoniae*. Based on these criteria, product sizes ranging from 8F-907 using the restriction enzymes *TSP509I* and *Hpy166II* were received.

For PCR reactions, only forward primer 8F was labeled with Carboxyfluorescein (FAM) as *in silico* analysis revealed that labeling of reverse primer 907 would not lead to additional discrimination. *In silico* fragment lengths of common nasopharyngeal species using the above criteria were received using Mica (http://mica.ibest.uidaho.edu/trflp.php), assuming that experimental results will differ by about 1%.

### DNA Extraction from Bacterial Cultures and PCR Amplification

To receive the *in vitro* terminal fragment (TF) lengths (compared to *in silico* as above) of common nasopharyngeal species, chromosomal DNA was extracted from *Streptococcus mitis, S. pseudopneumoniae, S. pneumoniae, S. oralis, S. gordonii, S. pyogenes, S. sanguinis, Pseudomonas aeruginosa, Rhodococcus equi, Corynebacterium pseudodiphtheriticum, Staphylococcus epidermidis, S. aureus, Haemophilus influenzae, Moraxella catarrhalis* and *Klebsiella pneumoniae* as previously described [11], [12]. PCR reactions were performed in a total volume of 50 µl using 1×fast start Taq reaction buffer, 1.5 mM MgCl2, 0.2 mM dNTPs, 1 µM of primer 8F FAM-AGA GTT TGA TCC TGG CTC AG and 1 µM of primer 907R CCG TCA ATT CMT TTG AGT TT, 0.2 µl of Fast start Taq Polymerase (Roche, Rotkreuz, Switzerland) and 2 µl of extracted DNA (2 ng/µl). PCR cycling conditions included a heat activation step at 95°C for 6 min, 35 cycles of denaturation at 95°C (30 s), annealing at 59°C (30 s) and elongation at 71°C (2 min) and a final elongation step at 71°C for 7 min. PCR products were purified using the Promega Wizard (Promega, Madison, WI).

### T-RFLP Digestion and Detection Limits

Purified PCR products were checked for correct amplification size and purity using the Agilent 2100 bioanalyzer (Agilent Technologies, Palo Alto, CA) with the DNA 7500 LabChip kit. The enzymatic digestions were performed in a total volume of 20 µl using 40 ng of the amplified and purified PCR product, 1 µl NEB Reaction Buffer (New England Biolabs, Ipswich, MA) 1 (for digestion with *Tsp509I*) or 4 (*Hpy166II*) and 0.5 µl of the restriction enzymes *Tsp509I* or *Hpy166II*. Digestion conditions were 3 h at 65°C for *TSP509I* and 3 h at 37°C with 20 min enzyme inactivation at 65°C for *Hpy166II*, respectively. Digested products were purified and capillary electrophoresis was performed on an AB 3130 genetic analyzer (Applied Biosystems, Rotkreuz, Switzerland) as previously described [12]. Detection limits were tested for *Streptococcus pneumoniae, Haemophilus influenzae, Moraxella catarrhalis* and *Corynebacterium pseudodiphtheriticum* by amplifying serial dilutions.

### 16S *rRNA* T-RFLP of Nasopharyngeal Swabs from Infants with AOM

We processed nasopharyngeal swabs collected from children younger than 2 years within an on-going nationwide surveillance program during four winter seasons in Switzerland [13]. The presence of *S. pneumoniae* was verified by culture and *plyNCR*-PCR data generated in earlier studies [11], [12], [14]. PCR and 16S gene T-RFLP digestions with *Tsp509I* or *Hpy166II* were performed as described above. The resulting T-RFLP electropherograms were uploaded to the Peak Scanner Software v1.0 (Applied Biosystems, Carlsbad, CA). Background noise was eliminated by setting the cut off at 50 fluorescence units (FU). The resulting sizing table was exported and the data was prepared for uploading to the T-RFLP analysis expedited software (T-Rex), which included creating a ‘genemapper’ and label file [15], [16]. For the genemapper file, a different fluorophor was assigned to the values derived from *Hpy166II* (Green) as compared to *Tsp509I* (Blue), which allowed the combined analysis of both enzymes for each sample at the same time. For processing the data with T-Rex, no additional filter for noisy peaks was set but clustering thresholds of 2.0 were used to align the TFs.

### Cloning and Sequencing

Purified PCR products were cloned into the pGEM-T Easy Vector (Promega) as per manufacturer's instructions. 20 colonies per ligation were picked at random. Plasmid DNA was purified using the Qiagen Plasmid Miniprep Kit and sequenced as described [6]. This protocol still results in longer reads as compared to pyrosequencing protocols and therefore allows precise assignment of bacterial taxa. Resulting sequences were submitted to GenBank (accession numbers JQ745789–JQ746034).

### Statistical Analysis

For comparing TF mean richness (mean number of TFs within all samples) between different environments, we performed unpaired t-test using Statview software defining significance if the two tailed *p*-value was <0.05. For the additive main effects and multiplicative interaction model (AMMI) of T-Rex, data included was based on TF presence, but not height or peak area [15], [16]. AMMI plot was derived by R as suggested by T-Rex. The function “mantel” in R was used to find the Mantel statistic as a matrix correlation between two dissimilarity matrices derived by the two enzymes.

## Results

### *In silico* Design of Terminal Restriction Fragment Length Polymorphism (T-RFLP) for the Nasopharynx

We first downloaded the complete 16S *rRNA* sequences of 38 bacterial strains characteristic for the nasopharynx based on previous studies from the ncbi webpage (www.ncbi.nlm.nih.gov), which we sheared in 4 different primer pair groups ranging from 8F-907, 8F-1391, 341–907 and 341–1391 (*E. coli* numbering). All commercially available restriction enzymes (n = 189) were then tested for optimal discrimination of these 16S *rRNA* gene sequences. The groups 8F-907 and 8F-1391 were found to be superior and primers for 8F-907 were subsequently designed. We then manually inspected the top 10 restriction enzymes for the additional ability to discriminate between *S. pneumoniae* and other members of the Mitis group streptococci but also within *Haemophilus* spp. and *Staphylococcus* spp. (Table S1). We identified 2 enzymes (*Tsp509I* and *Hpy166II)*, fulfilling these criteria which we subsequently tested in the laboratory.

### *In vitro* Evaluation of the Restriction Enzymes *Tsp509I* and *Hpy166II*

For a proof of principle, we subsequently verified *in vitro* 14 of the 38 species that had been examined by *in silico* analysis. The strains represented the following taxa: *Corynebacteriaceae*, *Strepococcaceae*, *Staphylococcaceae*, *Haemophilus influenzae, Moraxella catarrhalis* and *Klebsiella pneumoniae* (Table 1). Results showed that TF sizes calculated *in silico* differed consistently about 1% of the predicted *in vitro* values (Table 1). The detection limit of the in vitro T-RFLP experiment was about 30 genome copies µl^−1^ with the exception of *H. influenzae* and *M. catarrhalis* (100 copies µl^−1^; data not shown).

**Table 1:** Predicted and observed terminal restriction fragments (TFs) in bp after separate digestion with *TSP509I* and *Hpy166I*.

Sample species name	*TSP509I*		*Hpy166I*	
	*In silico*	*In vitro*	*In silico*	*In vitro*
*Streptococcus mitis*	207	204	470	465
*Streptococcus pseudopneumoniae*	671	665	470	465
*Streptococcus pneumoniae*	671	664	206	202
*Streptococcus gordonii*	186	195	480	475
*Streptococcus pyogenes*	195	189	804	795
*Streptococcus sanguinis*	174	169	472	467
*Pseudomonas aeruginosa*	547	538	793	784
*Rhodococcus equi*	523	515	79	73
*Corynebacterium pseudodiphtheriticum*	524	516	415	410
*Staphylococcus epidermidis*	558	549	805	794
*Staphylococcus aureus*	558	549	472	465
*Haemophilus influenzae*	469	463	798	790
*Moraxella catarrhalis*	541	535	788	780
*Klebsiella pneumoniae*	549	540	796	784

TFs were received using the labeled 8F primer.

A differentiation between *S. pneumoniae* and other members of Mitis group streptococci could also be achieved (Table 1). However, the restriction enzyme *TSP509I* alone could not differentiate between *S. pneumoniae* and *S. pseudopneumoniae.* This could only be achieved when results of *TSP509I and Hpy166I* were analyzed in combination (Table 1).

### Overall TF Richness and Taxonomic Assignment

**Figure 1:**
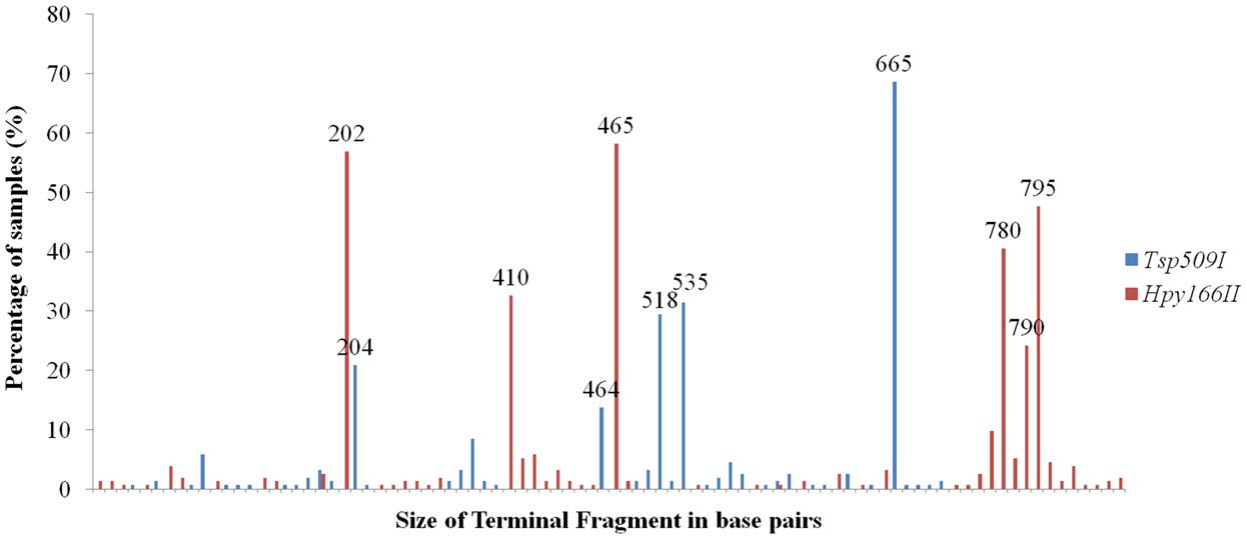
Terminal fragment (TF) prevalence in nasopharyngeal swabs from 153 infants with acute otitis media. Shown are the TF frequencies (in %) for the two different restriction enzymes (blue for *Tsp509I* and red for *Hpy166II*).

16S *rRNA* T-RFLP was performed for 153 nasopharyngeal samples from infants with otitis media using the two different restriction enzymes. Overall only 5 (using *Tsp509I*) and 6 (*Hpy166II*) TFs achieved a prevalence of more than 10% (Figure 1). Overall, the mean TF richness was 2.3 TFs (±1.5) (*Tsp509I*) and 3.5 TFs (±2.0) (*Hpy166II*) for the 153 samples analyzed and we received sample heterogeneity values of 17.0 (*Tsp509I*) and 13.2 (*Hpy166II*). We then chose 15 nasopharyngeal samples covering all TFs with a prevalence >6% for subsequent cloning and sequencing for taxa assignment. A total of 245 sequences of approximately 900 bp were received of which 232 could be assigned to a TF (Table 2). All TFs with a prevalence >6% could be identified with DNA sequencing allowing a sufficient description of bacterial community changes for the most important bacterial taxa. *Streptococcus* spp., *H. influenzae*, *Moraxella* spp. *Corynebacterium* spp. and *Staphylococcus* spp. were identified most frequently (Table 2).

**Table 2:** DNA sequencing and subsequent taxa assignments of the most prevalent *Tsp509I* terminal fragments (TFs) derived from 15 samples with different T-RFLP patterns.

*In silico* TF in bp	*In vitro* TF in bp	Taxa assignation	Number of samples with TF (%)	Number of DNA Sequences	
671	665	*Streptococcus* spp. 1*	105 (68.6)	84	
541	535	*Moraxella* spp. 1	48 (31.4)	7	
524	518	*Corynebacterium* spp.	45 (29.4)	32	
207	204	*Streptococcus* spp. 2*	32 (20.9)	14	
469	464	*Haemophilus influenzae*	21 (13.7)	3	
358	352	*Neisseria* spp.	13 (8.5)	8	
153 & 156	148	*Moraxella* spp. 2 *& Sphingobacterium* spp.	9 (5.9)	3 & 14**	
558	550	*Staphylococcus* spp.	7 (4.6)	8	
501	496	*Prevotella* spp.	5 (3.3)	5	
195	190	*Streptococcus pyogenes*	5 (3.3)	4	
561	552	*Gemella* spp.	4 (2.6)	1	
591	583	*Granulicatella* spp.	4 (2.6)	3	
551	544	*Stenotrophomonas maltophilia*	3 (2.0	8	
190	185	*Chrysebacterium* spp. 1	3 (2.0)	6	
702	697	*Chrysebacterium* spp. 2	2 (1.3)	6	
204	199	*Microbacterium* spp. 1	2 (1.3)	14	
374	371	*Fusobacterium* spp.	2 (1.3)	1	
169	167	*Turicella* spp.	1 (0.7)	6	
549	540	*Klebsiella* spp.	1 (0.7)	1	
645	640	*Propionibacterium* spp.	1 (0.7)	3	
209	206	*Porphyromonas* spp.	1 (0.7)	1	
529	524	*Microbacterium* spp. 2	1 (0.7)	1	
TOTAL			153 (100)	233	

* *Streptococcus pneumoniae* and *pseudopneumoniae* are included in *Streptococcus* spp. 1 while other members of the Mitis group are included in *Streptococcus* spp. 2. Using the second restriction enzyme (*Hpy166II*) *Streptococcus* spp. 1 can be further divided into *S. pneumoniae* and *S. pseudopneumoniae* (see text).

** Two different bacterial assignments were retrieved for TF with 148 base pairs (bp).

### Correlation of Distance Matrices and Comparison with 454 Pyrosequencing and Pneumococcal Culture Data

**Figure 2:**
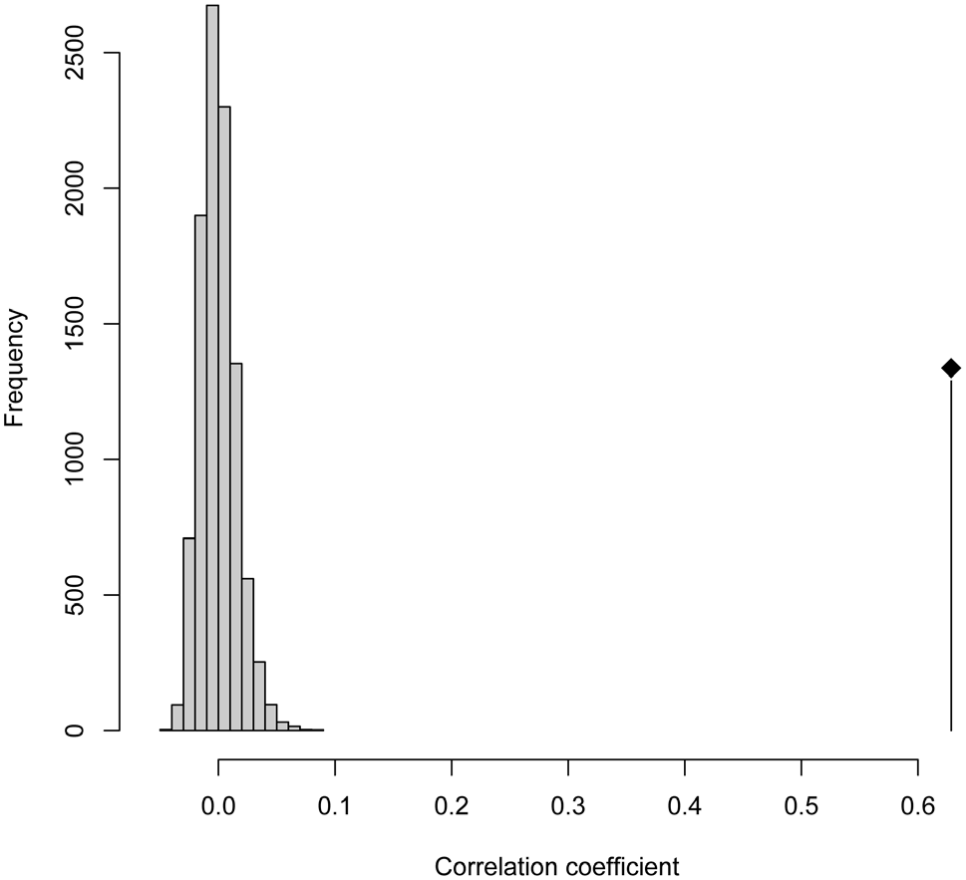
Mantel test comparing the distance matrices of *Tsp509I* and *Hpy166II*. The analysis includes the 2 different distance matrices derived of the T-RFLP data sets from 153 infants with acute otitis media. Illustrated is the frequency distribution of correlation coefficients generated from 10,000 permutations, and the location of the observed value. The correlation of the pair wise distance matrices between the two restriction enzymes is highly significant (*r* = 0.65, *P*<0.001) and therefore illustrates a comparable performance.

The performance of the 2 restriction enzymes was evaluated by creating pairwise distance matrices for all 153 samples and a subsequent mantel test. This revealed a high correlation between the two restriction enzymes (r = 0.65; Figure 2).

Comparing T-RFLP with recently published 454 pyrosequencing data revealed that *Moraxellaceae, Streptococcaceae, Pasteurellaceae, Staphylococcaceae* and *Corynebacteriaceae* were the most frequent bacterial families within the 153 samples with both methods [10]. However, the absolute frequencies of *Moraxellaceae* and *Pasteurellaceae*, were increased in the 454 compared to the T-RFLP data set. This therefore means that the two methods showed different absolute sensitivity values.

Pneumococcal culture detected *S. pneumoniae* in 80 samples (52.3%). In comparison, 79 *S. pneumoniae* could be identified with the T-RFLP method if results of *TSP509I* and *Hpy166I* were analysed in combination (100% specificity and 98.8% sensitivity).

### Demographic Factors Influencing the Mean Richness of TFs and Whole Microbial Community Comparison

**Figure 3:**
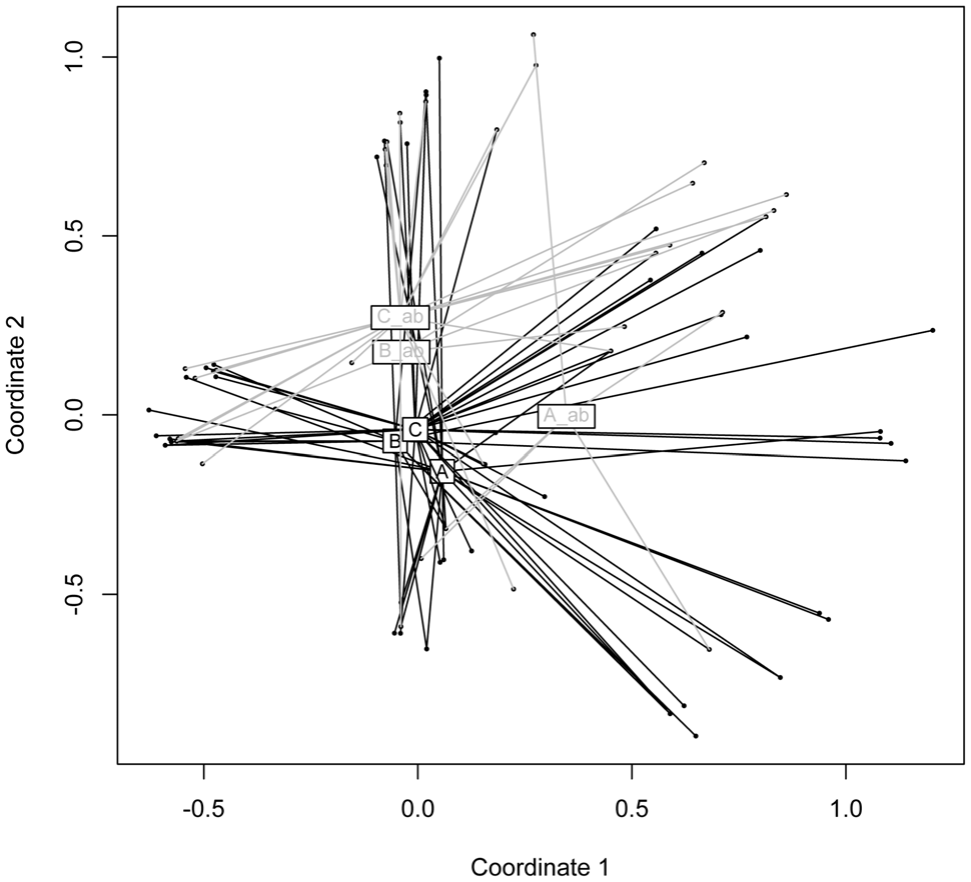
Additive Main Effects and Multiplicative Interaction Model (AMMI) analysis of bacterial T-RFLP data **sets.** Using the software T-Rex (T-RFLP analysis Expedited) [15], T-RFLP profiles were first analyzed individually and then grouped according to antibiotic exposure, vaccine season and vaccine status in the software R. The quadrates illustrate the centroids of the individual data points and represent the following: A) prevaccine era, not vaccinated, no antibiotic exposure. B) vaccine era, not vaccinated, no antibiotic exposure. C) vaccine era, vaccinated, no antibiotic exposure. A_ab) prevaccine era, not vaccinated, antibiotic exposure. B_ab) vaccine era, not vaccinated, antibiotic exposure. C_ab) vaccine era, vaccinated, antibiotic exposure. According to AMMI, antibiotic exposure (grey versus black) and vaccine season (A and A_ab versus others) significantly influences the microbiota (see text and Table 3).

Measuring mean richness differences within sex, day care and recent treatment with antibiotics did not reveal significant outcomes (Table 3). In contrast, PCV-7 vaccination status, year of sampling (prevaccine versus vaccine era) and age were identified as factors significantly influencing TF mean richness. Using an Additive Main Effects and Multiplicative Interaction Model (AMMI) revealed higher percentages of signal compared to noise interaction effects for prior antibiotic exposure (4.8 versus 1.0), samples from the vaccine era (3.4 versus 0.8) and age (1.5 versus 1.2). AMMI findings therefore conclude that antibiotic exposure and sampling in vaccine era mostly influenced the nasopharyngeal microbiota which was also illustrated in a Principal Component Analysis (PCA) plot (Figure 3).

**Table 3:** Terminal fragment (TF) richness and interaction effects resulting from two restriction enzymes (*Tsp509I* and *Hpy166II*).

		TF richness *Tsp509I*			TF richness *Hpy166II*			Interaction effects (%)*	
	n	Mean	SD	*p*	Mean	SD	*p*	Signal	Noise
**Sex**									
female	74	2.3	1.6	0.68	3.45	2.03	0.76	-0.1	1.4
male	79	2.2	1.4		3.48	2.10			
**Day care** **									
yes	54	2.3	1.4	0.69	3.52	1.96	0.78	0.4	1.3
no	89	2.2	1.5		3.38	2.08			
**Age**									
0	61	2.6	1.7	0.04	4.08	2.25	<0.01	1.5	1.2
1	92	2.1	1.3		3.05	1.83			
**Antibiotic treatment**									
yes	39	2.4	1.8	0.48	3.51	2.45	0.79	4.8	1.0
no	114	2.2	1.4		3.45	1.92			
**Seven valent pneumococcal polysaccharide vaccine (PCV-7)**									
yes	87	2.0	1.2	0.01	3.07	1.74	<0.01	0.8	1.3
no	66	2.6	1.7		3.98	2.33			
**Season**									
prevaccine era	31	3.1	2.1	<0.01	4.65	2.47	<0.01	3.4	0.8
vaccine era	122	2.0	1.2		3.2	1.8			

* Received by Additive Main Effects and Multiplicative Interaction Model (AMMI). Variation due to interaction effects can have a considerable range and reflect how similar or dissimilar the microbial communities are [15]. Therefore the more positive the difference between signal and noise, the more likely the parameters are influencing the communities [15], [16].

** No data concerning day care for 10 children.

These findings are in line with the pyrosequencing data published recently and therefore show the validity of the proposed T-RFLP method [10]. With sequencing, antibiotic exposure (p<0.005), and seasonality (prevaccine versus vaccine era) (p<0.05) were also shown to significantly influence the microbiota in the nasopharynx of children with AOM despite using different analysis tools. However, sequencing additionally revealed that PCV-7 vaccination status also has an influence (p<0.05).

### Demographic Factors Influencing Prevalence of Bacterial Community Members within Study Subjects

Subsequently, differences in the prevalence of the most frequent bacterial genera were calculated among the groups described above. Children with prior antibiotic exposure had a significantly lower prevalence of *Streptococcus* spp. TFs (*p* = 0.03) but a higher prevalence of *Haemophilus influenzae*. (Table 4) (*p* = 0.03). *Corynebacterium* spp. prevalence was lower for both, PCV-7 vaccination (*p* = 0.02) and samples originating from the vaccine era (*p* = 0.004).

**Table 4:** Prevalence of the most frequent bacterial taxa.

	Total	*Haemophilus influenzae* **		*Corynebacterium* spp.**		*Moraxella*spp.**		*Streptococcus*spp. (1)**		*Streptococcus*spp. (2)**	
	n	n (%)	*p*	n (%)	*p*	n (%)	*p*	n (%)	*p*	n (%)	*p*
**Sex**											
female	74	8 (11)	0.35	22 (30)	1	26 (35)	0.38	51 (69)	1	15 (20)	1
male	79	13 (16)		23 (29)		22 (28)		54 (68)		17 (22)	
**Day care** *											
yes	54	7 (13)	1	14 (26)	0.2	19 (35)	0.71	41 (76)	0.14	11 (20)	1
No	89	13 (15)		31 (35)		28 (31)		56 (63)		18 (20)	
**Age**											
0 years	61	9 (15)	0.81	22 (36)	0.15	22 (36)	0.37	37 (61)	0.11	16 (26)	0.22
1 years	92	12 (13)		23 (25)		26 (28)		68 (74)		16 (17)	
**Antibiotic treatment**											
Yes	39	10 (26)	0.03	7 (18)	0.1	10 (26)	0.43	21 (54)	0.03	11 (28)	0.25
No	114	12 (11)		38 (33)		38 (33)		84 (74)		21 (18)	
**Seven valent pneumococcal polysaccharide vaccine (PCV-7)**											
yes	87	13 (15)	0.64	19 (22)	0.02	28 (32)	0.86	57 (66)	0.38	17 (20)	0.69
no	66	8 (12)		26 (39)		20 (30)		48 (73)		15 (23)	
**Season**											
prevaccine era	31	5 (16)	0.77	16 (52)	0.004	10 (32)	1	23 (74)	0.52	9 (29)	0.22
vaccine era	122	16 (13)		29 (24)		38 (31)		82 (67)		23 (19)	

* No data concerning day care for 10 children.

** Data based on *Tsp509I* received TFs of 464 bp (*Haemophilus influenzae*), 518 bp (*Corynebacterium* spp.) 535 bp (*Moraxella* spp.), 665 bp (*Streptococcus* spp. 1) and 204 bp (*Streptococcus* spp. 2). *Streptococcus pneumoniae* and *pseudopneumoniae* are included in *Streptococcus* spp. 1 while other members of the Mitis group are included in *Streptococcus* spp. 2 (see Table 2).

Sequencing revealed an higher prevalence of *Pasteurellaceae* and a lower prevalence of *Streptococcaceae* for children with antibiotic exposure [10]. This was confirmative as well as the lower prevalence of *Corynebacteriaceae* with PCV-7 status and seasonality. However, sequencing additionally found many more commensals being influenced by antibiotic treatment and PCV-7 but their prevalence was small and fell beyond detection limit of the 16S gene T-RFLP. Their importance as persistent or transient colonizers has to be revealed in the future.

## Discussion

In this study, a novel 16S *rRNA* T-RFLP method was developed using 2 restriction enzymes (*TSP509I and Hpy166I)* able to discriminate colonizers of the nasopharynx most efficiently. Despite known high 16S *rRNA* gene sequence homology for members of the Mitis group, the enzymes were also able to discriminate among streptococcal species. So far, differentiation of *S. pneumoniae* from other Mitis group members was hampered by the close genetic homogeneity and horizontal gene transfer [17]. Analyzing 634 strains, Scholz *et al.* recently demonstrated a mutation in the 16S *rRNA* gene which reliably discriminated pneumococci from other members of the Mitis group [18]. This SNP is the corresponding cutting site for the two restriction enzymes *TSP509I* and *Hpy166I* and therefore enables this specific discrimination.

Within our study, we used the *T-REX* (T-RFLP analysis EXpedited) for data analysis [15]. This software has originally been designed for the analysis of T-RFLP data from soil communities but its applicability for studies of the human microbiome has recently been demonstrated [19]. This software offers two approaches for an automated alignment (binning) of peaks. We used the option which models the approach taken by the software program T-Align [20] and set the clustering threshold at 2.0. Setting an appropriate value for the clustering threshold is crucial for the evaluation of the TF richness. Setting it too low or too high may lead to an over- and underestimation of TF richness, respectively. This is a drawback of the methodology but newly developed, high throughput sequencing technologies face the same challenge as analyzing sequencing data also involves defining of Operational Taxonomic Units (OTU) based on an incidental cut off (most often 97% sequence identity) [21].

Another important factor for the optimal detection sensitivity is the choice of primers for the experiment. A recent study using the same forward but a different reverse primer revealed that T-RFLP was able to detect *S. aureus* when there were more than 30 genome copies µl^−1^ in 50 µl DNA extract from a nasal swab [22]. While we discovered a comparable detection limit for *Streptococcus* spp. and *Corynebacterium spp*., this was not the case for *Haemophilus influenzae* and *Moraxella* spp., probably due to a SNP present in the conserved region of the forward primer and therefore suboptimal PCR efficiency. Indeed, when comparing T-RFLP with 454 pyrosequencing data [10], *Haemophilus influenzae* and *Moraxella spp.* were less but *Corynebacterium spp.* and *Streptococcus spp.* more prevalent in the T-RFLP data set compared to sequencing. However, the relative changes among the groups were reproducible with the prevalence of *Haemophilus influenzae* being higher and *Corynebacterium* spp. being lower in the children exposed to antibiotic treatment and PCV-7, respectively. It is known that no primer pair is optimal for all members of a microbial community and the use of ambiguous primers, though there is a more optimal coverage *in silico*, does not necessarily improve PCR efficiency *in vitro* [23]. Additional reasons why received results may not always reflect the very correct bacterial composition of the clinical samples are for example the different copy numbers of the 16S *rRNA* gene within different bacterial members.

In our study, overall TF richness was low while sample heterogeneity was high compared to a recent study analyzing the oral communities [19]. Although it is known that the bacterial diversity in the nasopharynx is lower than in the oropharynx, the TF richness results may still seem surprisingly low. However, all the infants included in this study had acute otitis media which may have additionally reduced diversity compared to healthy children. In fact, most of these infants may have presented with a bacterial infection which has previously shown to reduce overall diversity [24].

In order to show the validity of the proposed T-RFLP method we additionally investigated if the same factors were influencing the microbiota of the 153 samples as received by recently published 454 pyrosequencing data [10]. We hypothesized that although the detection limit and amplification protocols of the two methods are slightly different the outcome had to be same in order to show the robustness of the T-RFLP method. The two main factors influencing the composition of the nasopharyngeal flora were prior exposure to antibiotics and PCV-7 vaccination. However, 16s *rRNA* gene T-RFLP only identified seasonality (prevaccine versus vaccine era) and not PCV-7 status as a driving factor for the composition of the microbiota while 454 data showed an influence of both. Age, day care and sex did not show an influence on the microbiota which was in accordance with our previous study [10].

Finally, we investigated if PCV-7 vaccination status and seasonality and antibiotic treatment were affecting frequencies of individual bacterial members of the nasopharynx in concordance tot 454 pyrosequencing results.

As expected, *Haemophilus influenzae* showed higher and commensal streptococci a lower prevalence in infants with antibiotic exposure. In addition prevalence of *Corynebacterium spp.* was lower in infants vaccinated with PCV-7 which was also in accordance to previously described results [10].

In conclusion, this study describes the development and validation of a 16S *rRNA* T-RFLP method to characterize the nasopharyngeal microbiota. It is not as sensitive for *Haemophilus influenzae* and *Moraxella spp.* as a recently published 454 pyrosequencing approach, but, in contrast, is able to discriminate bacterial species within the Mitis group of streptococci as the designed primer pair spans the regions with the relevant SNP. Both approaches do therefore not show the ‘optimal’ resolution of the microbial communities but reach the same conclusion that the introduction of PCV-7 and prior antibiotic exposure had effects on the composition of the flora. If used complimentary and validated carefully, we therefore suggest that in ‘omics’ era there is still a place for well-established methods like T-RFLP as they are cost effective, rapidly performed and interpreted.

## Supporting Information

Table S1 Predicted digestion of the 10 restriction enzymes resulting in the highest diversity. Terminal fragments were received using the forward sequences starting from position 8 (*E. coli* numbering). Differentiation among *Haemophilus* sp., *Staphylococcus* sp. and *Streptococcus* sp. is indicated in red for the two chosen enzymes.
(DOCX)
