# Supplementary material for: 16S rRNA Terminal Restriction Fragment Length Polymorphism for the Characterization of the Nasopharyngeal Microbiota
Source: PLoS One. 2012 Dec 20;7(12):e52241. doi: 10.1371/journal.pone.0052241 (PMC3527403; doi:10.1371/journal.pone.0052241)
Supplement: Table S1 — Predicted digestion of the 10 restriction enzymes resulting in the highest diversity. Terminal fragments were received using the forward sequences starting from position 8 (E. coli numbering). Differentiation among Haemophilus sp., Staphylococcus sp. and Streptococcus sp. is indicated in red for the two chosen enzymes. (DOCX) [file pone.0052241.s001.docx]

**Supplementary table 1**

| Bacterial species | Strain name | ***Sse9I*** | ***Hpy8I*** | ***AccII*** | ***AluI*** | ***AspLEI*** | ***AspS9I*** | ***BshFI*** | ***BsiSI*** | ***Bst4CI*** | ***MspA1I*** |
| --- | --- | --- | --- | --- | --- | --- | --- | --- | --- | --- | --- |
|  |  | ***Tsp509I*** | ***Hpy166I*** | ***BstUi*** |  | ***HhaI*** | ***Sau96I*** | ***HaeIII*** | ***MspI*** |  |  |
| *Acinetobacter baumannii* | Type | **616** | **468** | **389** | **72** | **207** | **82** | **253** | **80** | **882** | **515** |
| *Acinetobacter johnsonii* | DQ864703 | **616** | **468** | **389** | **72** | **207** | **198** | **253** | **491** | **882** | **515** |
| *Acinetobacter lwoffii* | DQ289068 | **577** | **467** | **388** | **71** | **206** | **189** | **199** | **490** | **881** | **514** |
| *Chlamydophila pneumoniae* | Type | **2** | **815** | **403** | **248** | **734** | **212** | **418** | **504** | **140** | **525** |
| *Corynebacterium pseudodiphtheriticum* | X84258 | **524** | **415** | **221** | **232** | **365** | **168** | **67** | **161** | **450** | **221** |
| *Corynebacterium tuberculostearicum* | Type | **526** | **292** | **223** | **219** | **367** | **66** | **67** | **163** | **452** | **495** |
| *Corynebacterium xerosis* | AF024653 | **525** | **85** | **223** | **73** | **668** | **66** | **67** | **165** | **61** | **223** |
| *Fusobacterium nucleatum or canifelinum* | Type | **62** | **56** | **110** | **131** | **212** | **323** | **285** | **282** | **455** | **498** |
| *Fusobacterium periodonticum* | FJ471665 | **374** | **56** | **96** | **189** | **198** | **309** | **271** | **268** | **116** | **484** |
| *Haemophilus influenzae* | AY613486 | **469** | **798** | **170** | **75** | **364** | **324** | **206** | **496** | **61** | **520** |
| *Haemophilus parainfluenzae* | AY365450 | **621** | **479** | **170** | **75** | **364** | **196** | **206** | **496** | **61** | **520** |
| *Haemophilus pittmaniae* | Type | **623** | **481** | **172** | **76** | **366** | **198** | **208** | **311** | **61** | **522** |
| *Klebsiella pneumoniae* | ATCC13883T | **549** | **796** | **393** | **74** | **371** | **194** | **39** | **494** | **155** | **433** |
| *Moraxella catarrhalis* | Type | **541** | **788** | **385** | **72** | **560** | **314** | **249** | **486** | **927** | **510** |
| *Mycobacterium fortuitum* | AF480580 | **523** | **415** | **173** | **197** | **175** | **66** | **67** | **159** | **449** | **221** |
| *Mycoplasma pneumoniae* | AF132740 | **350** | **394** | **390** | **146** | **226** | **192** | **254** | **545** | **473** | **604** |
| *Neisseria meningitidis* | AF310566 | **355** | **798** | **395** | **75** | **213** | **196** | **206** | **496** | **477** | **465** |
| *Neisseria sp.* | DQ409137 | **355** | **798** | **395** | **75** | **373** | **196** | **206** | **496** | **477** | **520** |
| *Neisseria sp.* | EU794238 | **355** | **798** | **395** | **75** | **373** | **196** | **206** | **496** | **477** | **468** |
| *Prevotella melaninogenica* | AY323525 | **503** | **96** | **119** | **32** | **104** | **265** | **266** | **99** | **141** | **522** |
| *Prevotella nigrescens* | AF414834 | **503** | **96** | **119** | **247** | **104** | **265** | **165** | **99** | **331** | **522** |
| *Prevotella salivae* | Type | **501** | **94** | **117** | **32** | **102** | **263** | **264** | **97** | **329** | **520** |
| *Propionibacterium acnes* | AB10847 | **645** | **421** | **227** | **175** | **671** | **66** | **67** | **165** | **455** | **498** |
| *Pseudomonas aeruginosa* | TDSM50071 | **547** | **793** | **392** | **63** | **370** | **39** | **41** | **146** | **321** | **92** |
| *Rhodococcus equi* | Type | **523** | **79** | **221** | **169** | **665** | **215** | **217** | **159** | **61** | **221** |
| *Staphylococcus aureus* | Type | **558** | **472** | **236** | **74** | **238** | **331** | **310** | **155** | **208** | **527** |
| *Staphylococcus caprae* | Type | **558** | **805** | **236** | **74** | **238** | **331** | **310** | **155** | **198** | **527** |
| *Staphylococcus cohnii* | Type | **558** | **805** | **236** | **74** | **238** | **230** | **310** | **147** | **331** | **527** |
| *Staphylococcus epidermidis* | Type | **558** | **805** | **236** | **74** | **238** | **331** | **310** | **155** | **208** | **527** |
| *Streptococcus gordonii* | Type | **186** | **480** | **121** | **78** | **589** | **237** | **317** | **563** | **65** | **535** |
| **Supplementary table 1 continuation** | |  |  |  |  |  |  |  |  |  |  |
| *Streptococcus mitis* | Type | **207** | **470** | **111** | **73** | **579** | **227** | **307** | **83** | **476** | **525** |
| *Streptococcus parasanguinis* | Type | **673** | **179** | **113** | **67** | **581** | **229** | **309** | **555** | **478** | **527** |
| *Streptococcus pneumoniae* | Type | **671** | **206** | **111** | **73** | **579** | **227** | **307** | **553** | **476** | **525** |
| *Streptococcus pseudopneumoniae* | Type | **671** | **470** | **111** | **73** | **579** | **227** | **307** | **553** | **476** | **525** |
| *Streptococcus pyogenes* | AB023575 | **195** | **804** | **113** | **160** | **581** | **229** | **309** | **81** | **484** | **527** |
| *Streptococcus salivarius* | AY188352 | **209** | **472** | **113** | **74** | **581** | **229** | **309** | **555** | **478** | **527** |
| *Streptococcus sanguinis* | Type | **174** | **472** | **113** | **74** | **581** | **229** | **309** | **555** | **478** | **527** |
| *Veillonella dispar* | AF439639 | **571** | **826** | **115** | **75** | **590** | **277** | **212** | **302** | **148** | **540** |
